# Gnrh2 maintains reproduction in fasting zebrafish through dynamic neuronal projection changes and regulation of gonadotropin synthesis, oogenesis, and reproductive behaviors

**DOI:** 10.1038/s41598-021-86018-3

**Published:** 2021-03-23

**Authors:** Miranda Marvel, Berta Levavi-Sivan, Ten-Tsao Wong, Nilli Zmora, Yonathan Zohar

**Affiliations:** 1 Department of Marine Biotechnology, Institute of Marine and Environmental Technology, University of Maryland Baltimore County, Baltimore, MD 21202 USA; 2 Department of Animal Sciences, The Robert H. Smith Faculty of Agriculture, Food, and Environment, The Hebrew University of Jerusalem, 76100 Rehovot, Israel

**Keywords:** Sexual behaviour, Mutagenesis, Endocrine system and metabolic diseases

## Abstract

Restricted food intake, either from lack of food sources or endogenous fasting, during reproductive periods is a widespread phenomenon across the animal kingdom. Considering previous studies show the canonical upstream regulator of reproduction in vertebrates, the hypothalamic Gonadotropin-releasing hormone (Gnrh), is inhibited in some fasting animals, we sought to understand the neuroendocrine control of reproduction in fasted states. Here, we explore the roles of the midbrain neuropeptide, Gnrh2, in inducing reproduction via its pituitary prevalence, gonadotropin synthesis, gametogenesis, and reproductive outputs in the zebrafish model undergoing different feeding regimes. We discovered a fasting-induced four-fold increase in length and abundance of Gnrh2 neuronal projections to the pituitary and in close proximity to gonadotropes, whereas the hypothalamic Gnrh3 neurons are reduced by six-fold in length. Subsequently, we analyzed the functional roles of Gnrh2 by comparing reproductive parameters of a Gnrh2-depleted model, *gnrh2*^−/−^, to wild-type zebrafish undergoing different feeding conditions. We found that Gnrh2 depletion in fasted states compromises spawning success, with associated decreases in gonadotropin production, oogenesis, fecundity, and male courting behavior. Gnrh2 neurons do not compensate in other circumstances by which Gnrh3 is depleted, such as in *gnrh3*^−/−^ zebrafish, implying that Gnrh2 acts to induce reproduction specifically in fasted zebrafish.

## Introduction

Reduced feeding behaviors during the reproductive season is a common attribute in many organisms^1–3^. Numerous animals, including snakes^4–6^, birds^1,2,7,8^, marine mammals^9^, and others^10,11^ limit food intake or cease feeding altogether, due to endogenous cues or lack of environmental food sources, when they switch their energy priority to reproductive activities and parental care. In fishes especially, many species cease feeding for weeks or months at a time during the reproductive season^12^, a phenomena which is exemplified by the long fasting periods of migratory salmon and eel prior to spawning^13–15^, as well as the seasonally fasting winter flounder^16^ and tropical wolf fish^17^. Despite the prevalence of limiting food intake during reproduction and incubation, the neuroendocrine control of reproduction during environmentally relevant fasting conditions has not been well studied.

The gonadotropin-releasing hormone (Gnrh) neuropeptide system is canonically known as the master regulator of reproduction^18,19^. The Gnrh neuropeptides are widely conserved amongst vertebrates and so far, three forms of Gnrh have been discovered which are named based on their brain location and proposed function. Gnrh1 is found in the hypothalamus, and thought to be the main hypophysiotropic (reaching the median eminence or pituitary) form, stimulating the release of the gonadotropins, luteinizing hormone (Lh) and follicle-stimulating hormone (Fsh), from the pituitary^18,19^. These gonadotropins circulate through the bloodstream and subsequently induce steroid production, gametogenesis, and mating behaviors. Gnrh2 is located in the midbrain tegmentum and is the most conserved amongst vertebrates, although the major functions of this neuropeptide are still unknown. Gnrh3 is a teleost-specific form located in the forebrain, and in species where Gnrh1 has been evolutionarily lost, such as in zebrafish, Gnrh3 is also found in the hypothalamus and thought to be a major stimulator of gonadotropins and reproduction. In some animals, including murine and teleost models, the main hypophysiotropic Gnrh, whether it be Gnrh1 or Gnrh3, have been shown to be inhibited or reduced by anorexigenic signals such as increased neuropeptide Y (Npy, a feeding-inducing neuropeptide), decreased Adiponectin (a hormone produced from adipocytes and other tissues), and decreased Leptin (an adipocyte-associated hormone), in food-deprived conditions^20–23^. This suggests that in these animals, other neuropeptides may regulate reproduction during fasting periods. Although the reproductive roles of Gnrh1 and Gnrh3 have been thoroughly explored, the roles and functions of the midbrain Gnrh2 neuropeptide, especially in reproductive maintenance, is lacking, most likely due to the evolutionary loss of this neuropeptide in popular murine research models. Nonetheless, the ubiquitously conserved amino acid sequence of Gnrh2 in all species that possess it suggests an important biological role. Supportingly, research in numerous organisms including teleosts and mammals, show conserved roles of Gnrh2 exerting anorexic responses^24–26^, as well as the ability to stimulate gonadotropin release^27,28^ and reproductive behaviors^29–31^, granting Gnrh2 critical regulatory roles of inhibiting feeding and stimulating reproduction.

Remarkably, Gnrh2 studies in musk shrews, a mammal which, like humans, has retained this neuropeptide, show that Gnrh2 neurons exhibit a plastic response to food deprivation, with increased soma and projections to reproductive regulatory brain regions, such as the median eminence, after fasting^32^. Similar to the Gnrh2 response in musk shrews, Gnrh2 projections in zebrafish show a dynamic response to feeding conditions, where food deprivation induces an increase in Gnrh2 neuronal projections to the pituitary^33^. These findings indicate that Gnrh2 may be more reproductively relevant in animals undergoing fasting. Despite this conserved response in musk shrews and zebrafish, the exact mechanisms by which Gnrh2 exerts its reproductive function is yet unknown. Specifically, it is not known how Gnrh2 neurons respond to fasting conditions and whether Gnrh2 functions directly at the level of the pituitary to induce Lh/Fsh, gametogenesis, and reproduction output.

Therefore, here we examined the roles of Gnrh2 in maintaining gonadotropin synthesis and reproductive performance in fed and fasted conditions in the model organism zebrafish. Zebrafish, similar to other teleosts, contain two forms of Gnrh, Gnrh2 and Gnrh3, are able to reproduce after long periods of fasting, and are easy to generate transgenic and knockout models to study protein functions. Naturally, zebrafish undergo highly variable environmental conditions in the wild^34^ and can successfully spawn even after long periods of no food. Therefore, the existence of a redundant reproductive regulatory system which conveys the ability to continue spawning even in periods of low food availability may be an evolutionary advantage to zebrafish, although more detailed analysis on the wild feeding behaviors of zebrafish would need to confirm this. In our study, in order to characterize the plasticity of Gnrh neurons after fasting, we analyzed the neuronal projection patterns of Gnrh2 and Gnrh3 in pituitaries of fish undergoing fed and fasted states. We then examined the effects of Gnrh2 depletion, using a Gnrh2 knockout (*gnrh2*^−/−^) model, to analyze gonadotropin expression and protein synthesis, and associated gametogenesis and reproductive capacities over a fasting period. We were able to conclude that Gnrh2 displays a dynamic projection pattern over a fasting period and is a key component in maintaining multiple reproductive parameters after two weeks fasting. Further studies on the reproductive role of Gnrh2 during fasting in multiple species will give a better idea on the conserved nature of its reproductive relevance.

## Results

### Characterization of Gnrh projection patterns in pituitaries of fed and fasted zebrafish

**Figure 1:**
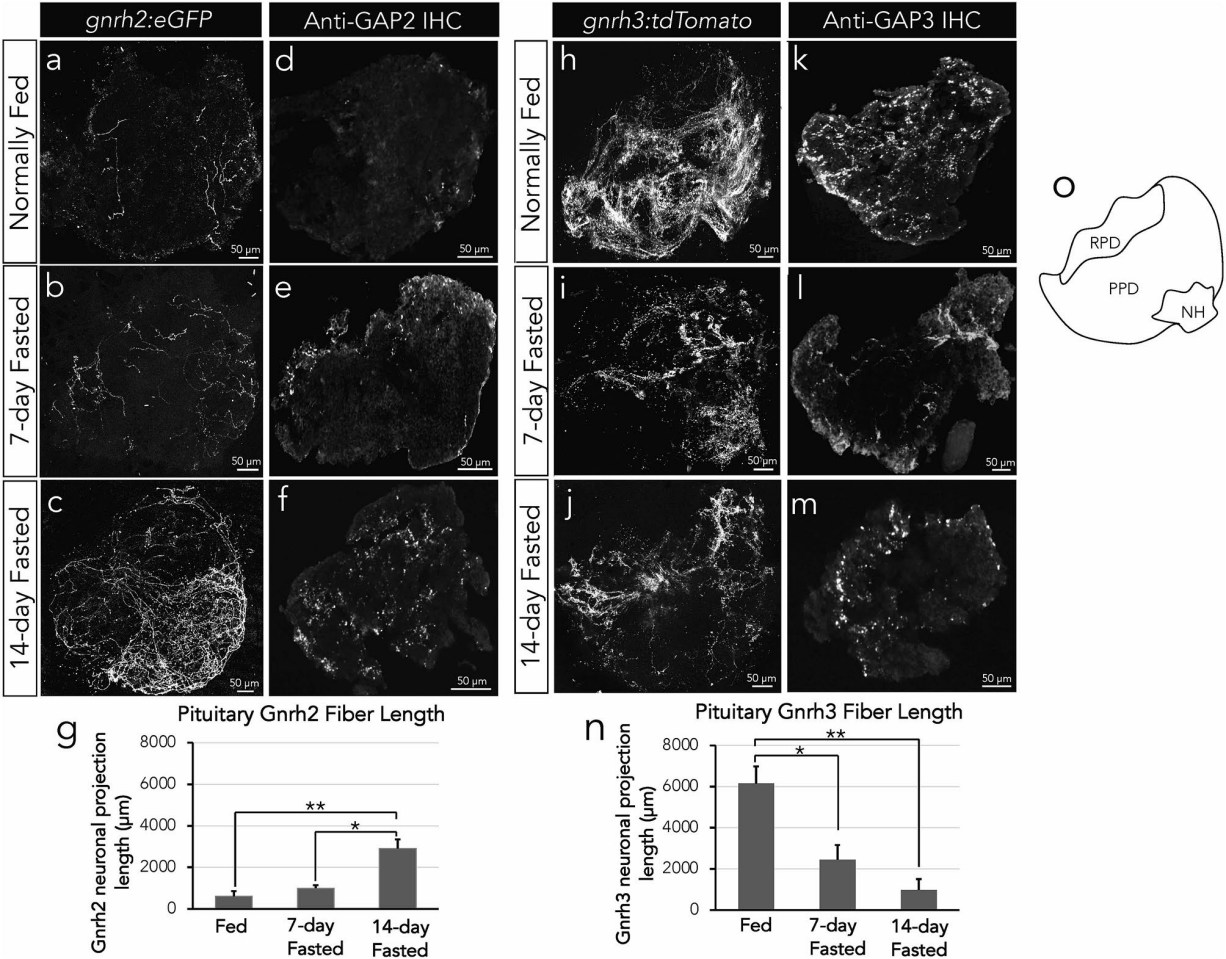
Confocal imagery of *tg(gnrh2:eGFP)* pituitaries in normally fed (a), 7-day fasted (b) and 14-day fasted (c) conditions. Antibody staining of the specific GAP2 region of Gnrh2 in pituitary cryosections of fed (d), 7-day fasted (e), and 14-day fasted zebrafish (d) with the quantification of Gnrh2 neuronal fiber lengths confirming increased Gnrh2 in 14-day fasted conditions (e). Confocal imagery of *tg(gnrh3:tdTomato)* pituitaries in normally fed (f) and 14-day fasted (h) conditions. Immunohistochemistry antibody staining of the specific GAP3 region of Gnrh3 in pituitary cryosections of fed (g) and 14-day fasted zebrafish (i) with the quantification of Gnrh3 neuronal fiber lengths confirming decreased Gnrh3 in 14-day fasted conditions (j). Pictoral representation of the pituitary orientation of images shown (k). NH, neurohypophysis; PPD, proximal pars distalis; RPD; rostral pars distalis. All data expressed as means ± S.E.M, n = 6 pituitaries per group, **P < 0.01, *P < 0.05.

In order to determine whether Gnrh neurons and associated protein presence change with different feeding conditions, we examined Gnrh2 and Gnrh3 neuronal fibers in the pituitaries of normally fed, 7-day fasted (fasting meaning the complete withholding of food), and 14-day fasted zebrafish (n = 6 pituitaries/group). We first examined and imaged zebrafish with previously developed and validated transgenically labelled Gnrh2 and Gnrh3 neurons^33^. We found an opposite response of Gnrh2 and Gnrh3 neurons to fasting. We visualized and quantified increased presence of Gnrh2:eGFP neurons in the pituitaries of 14-day fasted zebrafish (Fig. 1c) compared to fed (Fig. 1a) (P < 0.01) and 7-day fasted (Fig. 1b) (P < 0.05) zebrafish pituitaries, with more neurons projecting to the proximal pars distalis (PPD), where the gonadotropes are located. The length of the Gnrh2:eGFP neurons exhibited an almost four-fold increase compared to normally fed conditions after 14-days fasting (Fig. 1g) (P < 0.01). Contrastingly, there was a drastic reduction in Gnrh3:tdTomato neurons in zebrafish undergoing 14-day fasting (Fig. 1j), with decreased presence in the PPD and a six-fold decrease in neuronal projection lengths compared to fed conditions (Fig. 1h, n) (P < 0.01) and a three-fold decrease compared to 7-day fasting conditions (Fig. 1i, n) (P < 0.05). Antibody staining of specific GAP2 in Gnrh2 (Fig. 1d, e, f) and GAP3 in Gnrh3 (Fig. 1k, l, m) confirmed increased Gnrh2 protein in fasted pituitaries and decreased Gnrh3 protein in pituitaries of fasted zebrafish.

**Figure 2:**
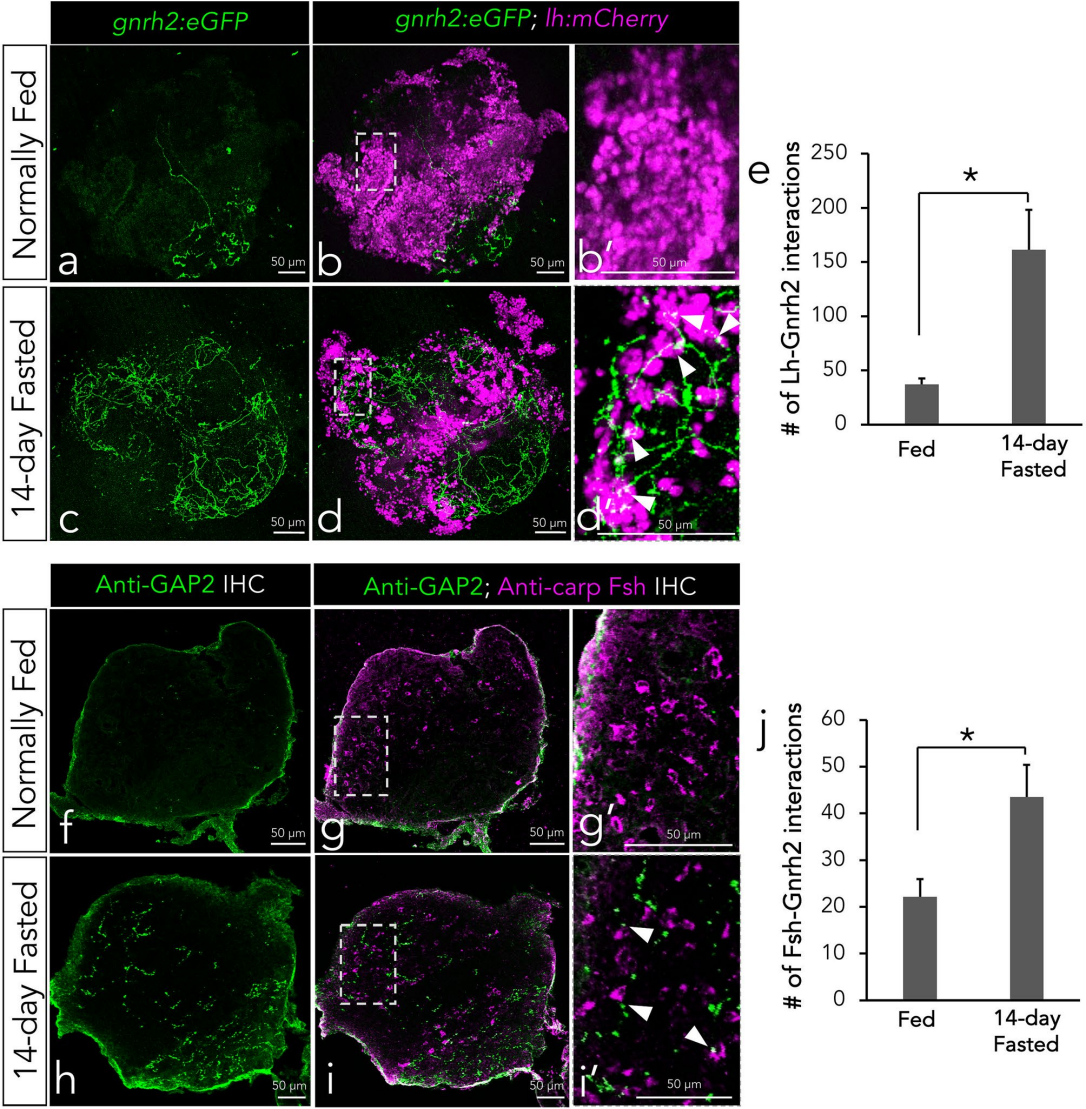
Confocal imagery of whole *tg(gnrh2:eGFP;lh:mCherry)* pituitaries in normally fed (a, b) and 14-day fasted zebrafish (c, d). B’ and d’ are magnifications of the boxed regions shown in b and d. Quantification of Lh cells with close Gnrh2 neuronal contact confirms increased interactions after 14-day fasting (E). Immunohistochemistry antibody staining of Gnrh2 and Fsh on pituitary cryosections of fish in normally fed (f, g) and 14-day fasted (h, i) conditions. G’ and i’ are magnifications of the squared regions shown in g and i. Quantification of Fsh cells with close Gnrh2 contact confirms increased interactions after 14-day fasting (j). ∆ indicate close contact of Gnrh2 neurons with gonadotropes. All data expressed as means ± S.E.M, n = 6 pituitaries per group, *P < 0.05.

Next, we analyzed the proximity of Gnrh2 neurons to Lh and Fsh gonadotropes (n = 6 pituitaries/group). Unlike mammals, some teleost hypophysiotropic neurons terminate directly on or proximal to gonadotrope cells in order to stimulate gonadotropin release. We used double transgenic *tg(gnrh2:eGFP;lh:mCherry)* to visualize Gnrh2 and Lh interactions in whole pituitaries (Fig. 2a-d) and found an almost four-fold increased abundance of Gnrh2:eGFP neurons in the adenohypophysis proximal to Lh cells in fasted zebrafish compared to fed fish (Fig. 2e) (P < 0.05). Since both Fsh and Gnrh2 are transgenically labelled with eGFP, we used antibody staining of pituitary cryosections to quantify Gnrh2 interactions with Fsh cells (Fig. 2f-i) and determined a two-fold higher number of Fsh cells in proximity to Gnrh2 neurons in fasted zebrafish compared to fed (Fig. 2j) (P < 0.05).

### Characterization of Gnrh expression profiles of fed and fasted zebrafish

**Figure 3:**
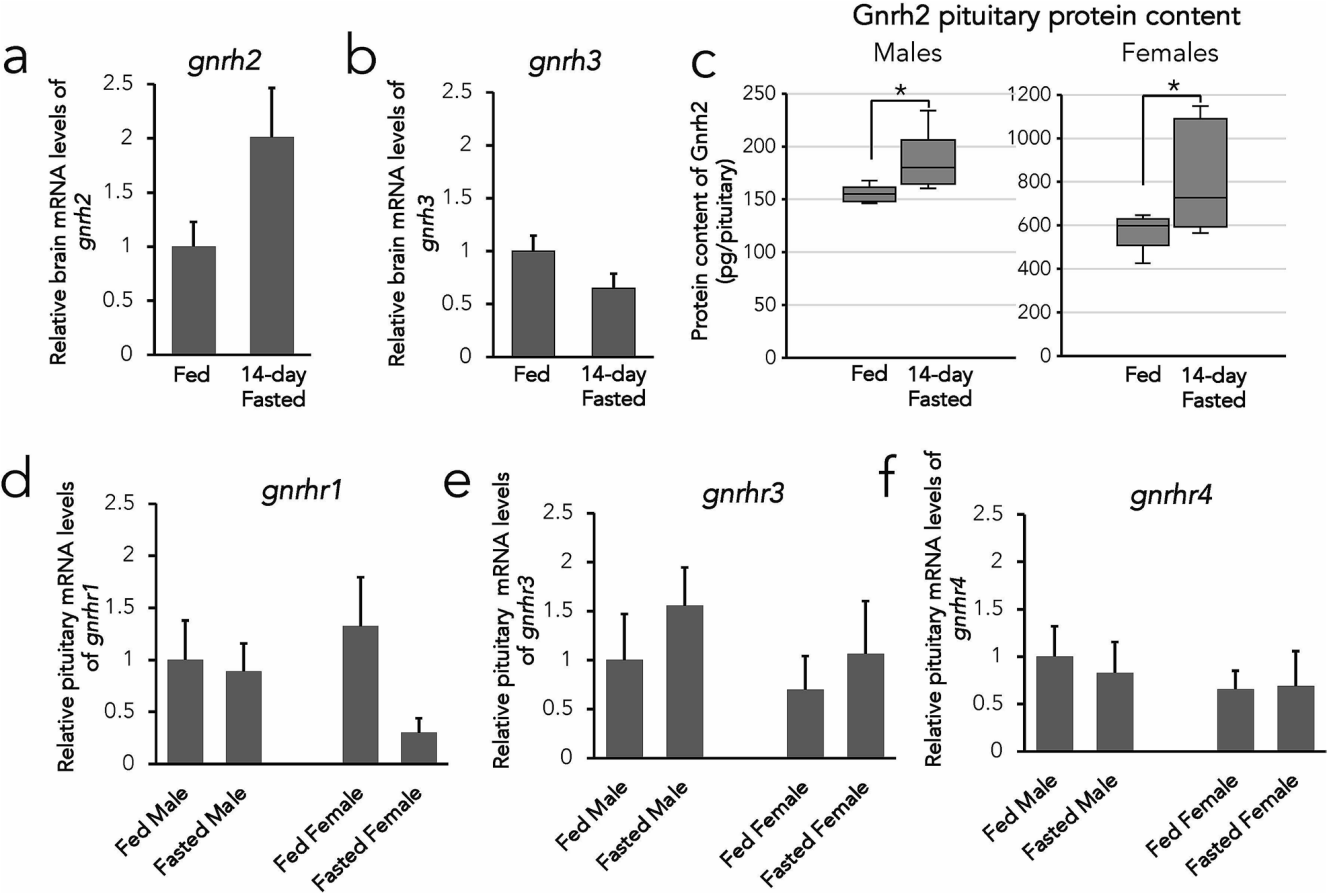
Expression profiles of the Gnrh system in fed and fasted conditions of wild-type zebrafish. Relative mRNA levels of *gnrh2* (a) and *gnrh3* (b) from brain tissues. Protein content of Gnrh2 in pituitaries from fed and 14-day fasted male and female wild-type zebrafish (c). Relative mRNA levels of *gnrhr1* (d), *gnrhr3* (e), and *gnrhr4* (f) in pituitaries from fed and fasted male and female wild-type zebrafish. All data expressed as means ± S.E.M, n = 8 pituitaries or brains per group, *P < 0.05.

We analyzed the expression pattern of the Gnrh ligands and receptors to comprehensively determine the expression changes occurring in fasted WT zebrafish (n = 8 pituitaries/group). In 14-day fasted zebrafish brains, there was no significant change of *gnrh2* expression (Fig. 3a) or *gnrh3* expression (Fig. 3b). In pituitary tissues, there was significantly higher Gnrh2 protein content from 14-day fasted male (P < 0.05) and female zebrafish (P < 0.05) compared to fed zebrafish (Fig. 3c). The levels of Gnrh receptor mRNAs *gnrhr1* (Fig. 3d), *gnrhr3* (Fig. 3e), and *gnrhr4* (Fig. 3f) exhibited no significant differences in mRNA levels in pituitaries from 14-day fasted zebrafish compared to fed zebrafish. There was no detectable *gnrhr2* expression in zebrafish pituitaries and therefore was not included in this study.

### Differential expression of Lh and Fsh in wild-type versus ***gnrh2***^−/−^ pituitaries undergoing different feeding conditions

**Figure 4:**
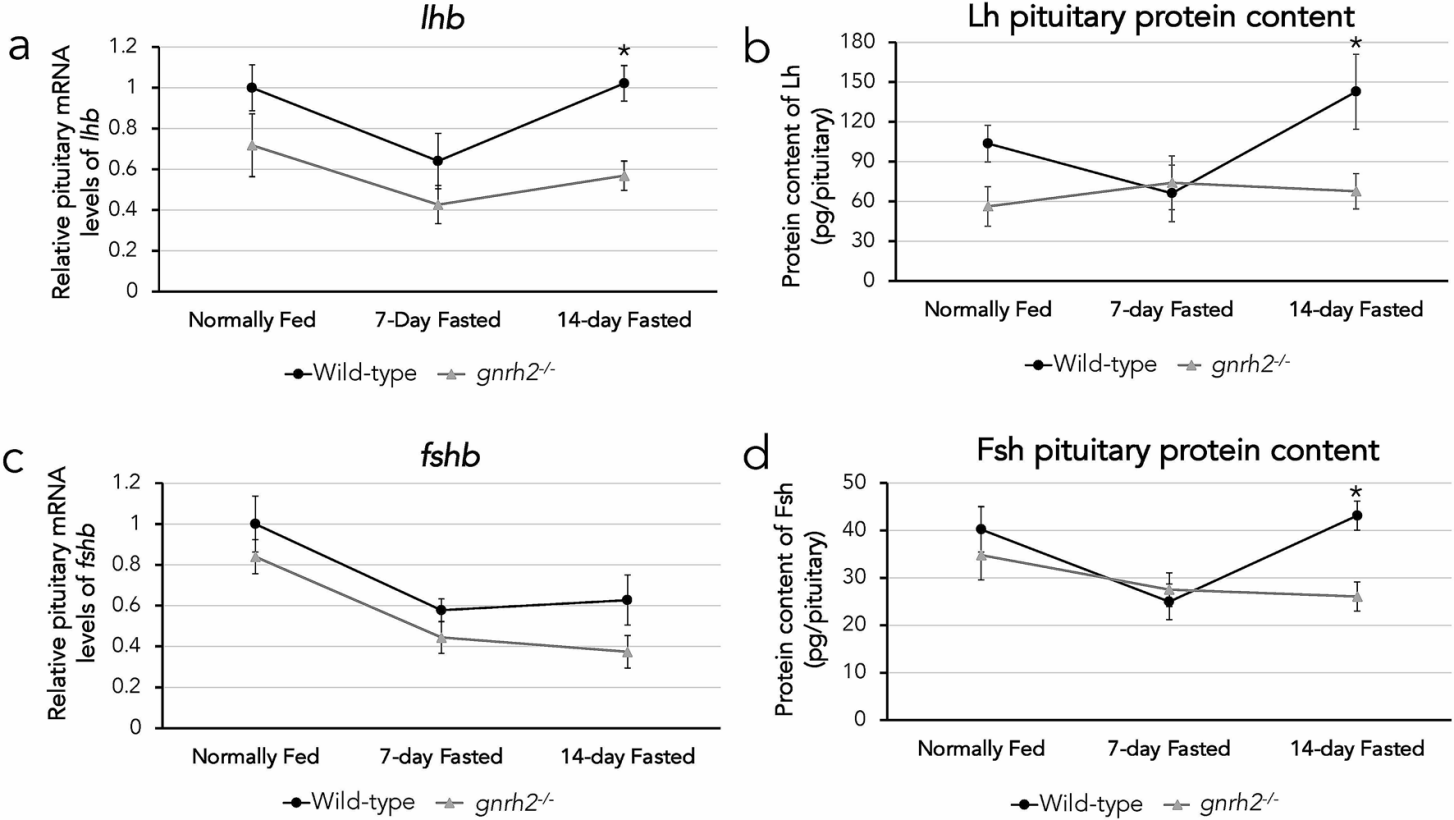
Expression differences of Lh and Fsh in wild-type and *gnrh2*^−/−^ zebrafish. Relative mRNA levels of *lhb* (a) and *fshb* (c) from pituitaries of wild-type ● and *gnrh2*^−/−^ ∆ zebrafish undergoing normally fed, 7-day fasting, or 14-day fasting conditions. Protein content of Lh (b) and Fsh (d) from pituitaries of wild-type ● and *gnrh2*^−/−^ ∆ zebrafish undergoing normally fed, 7-day fasting, or 14-day fasting conditions. All data expressed as means ± S.E.M, n = 8 pituitaries per group, *P < 0.05.

We next examined the effect of the loss of Gnrh2 on Lh and Fsh expression in fed and fasted conditions by comparing mRNA and protein levels from WT and *gnrh2*^−/−^ pituitaries (n = 8 pituitaries/group). There were no significant differences of *fshb* (Fig. 4a) and *lhb* (Fig. 4c) transcript levels between WT and KO pituitaries in the fed or 7-day fasted conditions. Although there were no significant differences in *fshb* mRNA levels between WT and *gnrh2*^−/−^ 14-day fasted pituitaries (Fig. 4c), there was significantly decreased Fsh protein content in *gnrh2*^−/−^ pituitaries compared to WT in zebrafish undergoing 14 day fasting (Fig. 4d) (P < 0.05). Additionally, the mRNA levels of *lhb* (Fig. 4a) and protein content of Lh (Fig. 4b) were significantly lower in *gnrh2*^−/−^ pituitaries compared to WT at 14 days of fasting (P < 0.05). Overall, the absence of Gnrh2 was associated with decreased Lh and Fsh protein in pituitaries after 14-days fasting. The intra-assay coefficient of variability (CV) for Lh and Fsh ELISA plates were 6.35% and 2.56% respectively.

### Gametogenesis comparison of wild-type versus ***gnrh2***^−/−^ zebrafish undergoing different feeding conditions

**Figure 5:**
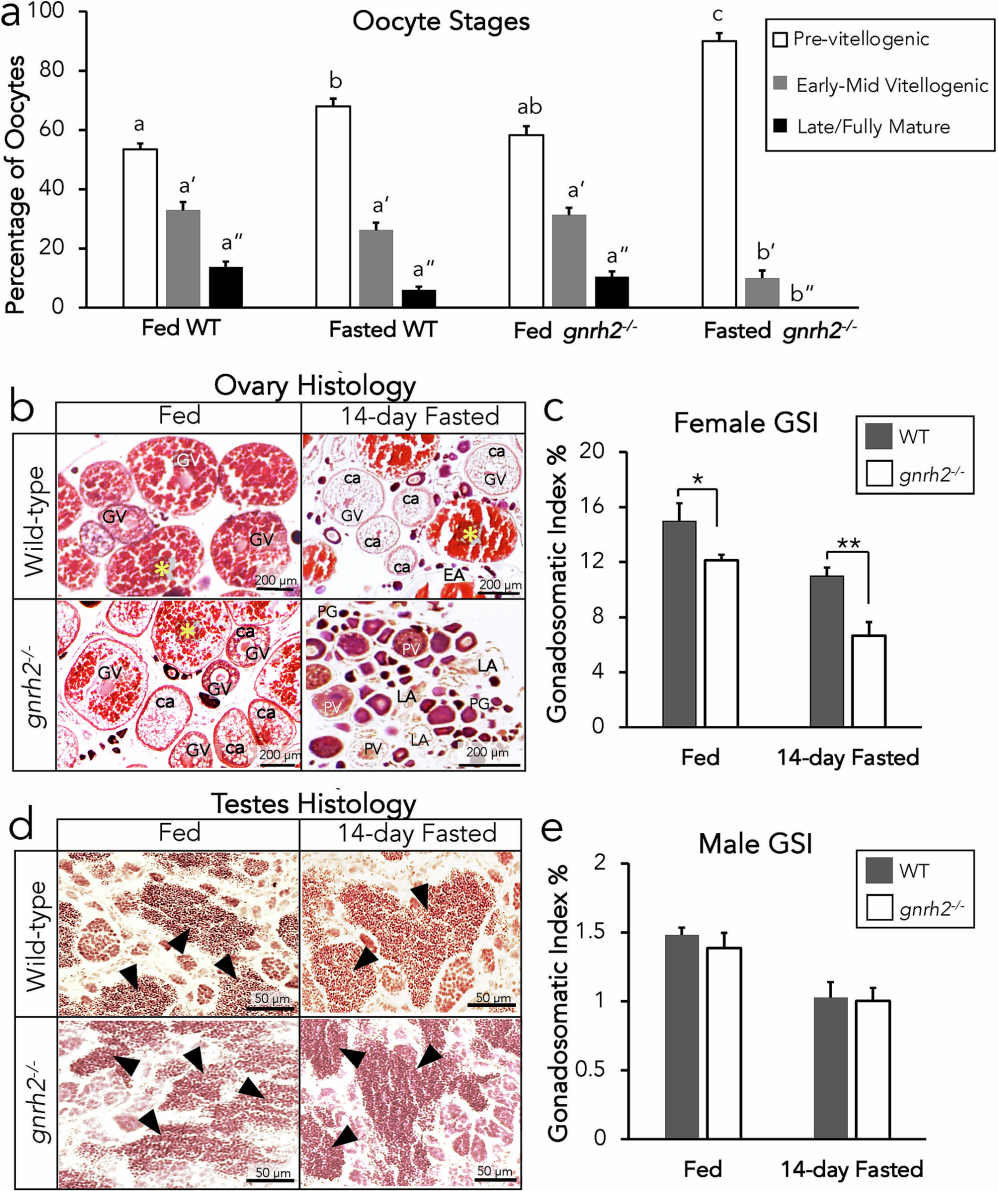
Gametogenesis analysis of knockout and WT zebrafish undergoing fed and fasted conditions. Percentage of oocytes at pre-vitellogenic, early-mid vitellogenic, and late/fully mature stages of WT and *gnrh2*^−/−^ ovaries after fed and fasted conditions demonstrate significantly fewer late/fully mature oocytes in *gnrh2*^−/−^ in fasted conditions (a). Histological sections of ovaries with labelled germinal vesicles (GV), cortical alveoli (ca), pre-vitellogenic oocytes (PV), and primary-growth follicles (PG), demonstrating fully mature oocytes (yellow star *) present in fed and fasted WT ovaries, but only present in fed *gnrh2*^−/−^ ovaries (b). GSI % quantification indicates reduced GSI in *gnrh2*^−/−^ undergoing fed and fasted conditions (c). Histological sections of testes demonstrate mature spermatozoa ▲ in fed and fasted WT and *gnrh2*^−/−^ males (d) with GSI % quantification indicating no difference in genotypes undergoing fed and fasted conditions (e). All data expressed as means ± S.E.M, n = 10 gonads per group, *P < 0.05, **P < 0.01, different letters indicate significant differences (P < 0.05) from each other (statistical comparisons made only between similarly-staged oocytes which are characterized by quote marks: no mark = pre-vitellogenic, ‘ = early-mid vitellogenic, ‘’ = late/fully mature).

We examined the effects of the loss of Gnrh2 on gametogenesis of males and females undergoing fed and fasting conditions through comparing wild-type and *gnrh2*^−/−^ oogenesis, spermatogenesis, and GSI (n = 8 gonads/group). In both WT and *gnrh2* mutants, ovaries exhibited increased levels of pre-vitellogenic oocytes (at the oogonia, primary growth, and cortical alveoli stages) and decreased late maturing/fully mature oocytes after 14 days of fasting (Fig. 5a). Only *gnrh2*^−/−^ females exhibited significantly decreased fully mature oocytes after 14 days of fasting with no visible mature oocytes in histological sections (Fig. 5b) (P < 0.05), whereas WT still contained some fully mature oocytes in fasted ovaries (Fig. 5b). Residues of late atretic follicles were seen mostly in *gnrh2*^−/−^ ovarian histological sections after 14-days of fasting, with 14-day fasted WT ovaries showing some evidence of oocytes undergoing early atresia (Fig. 5b). Additionally, *gnrh2*^−/−^ females exhibited 20% decreased GSI in fed conditions (P < 0.05), and after 14 days of fasting, exhibited a greater difference with 40% decreased GSI compared to WT females (Fig. 5c) (P < 0.01). WT and *gnrh2*^−/−^ males exhibited fully mature spermatozoa in both fed and 14-day fasted conditions (Fig. 5d) and, unlike females, showed no differences in GSI in fed or fasted conditions (Fig. 5e).

### Reproductive analysis of wild-type versus ***gnrh2***^−/−^ and ***gnrh3***^−/−^ zebrafish undergoing different feeding conditions

**Figure 6:**
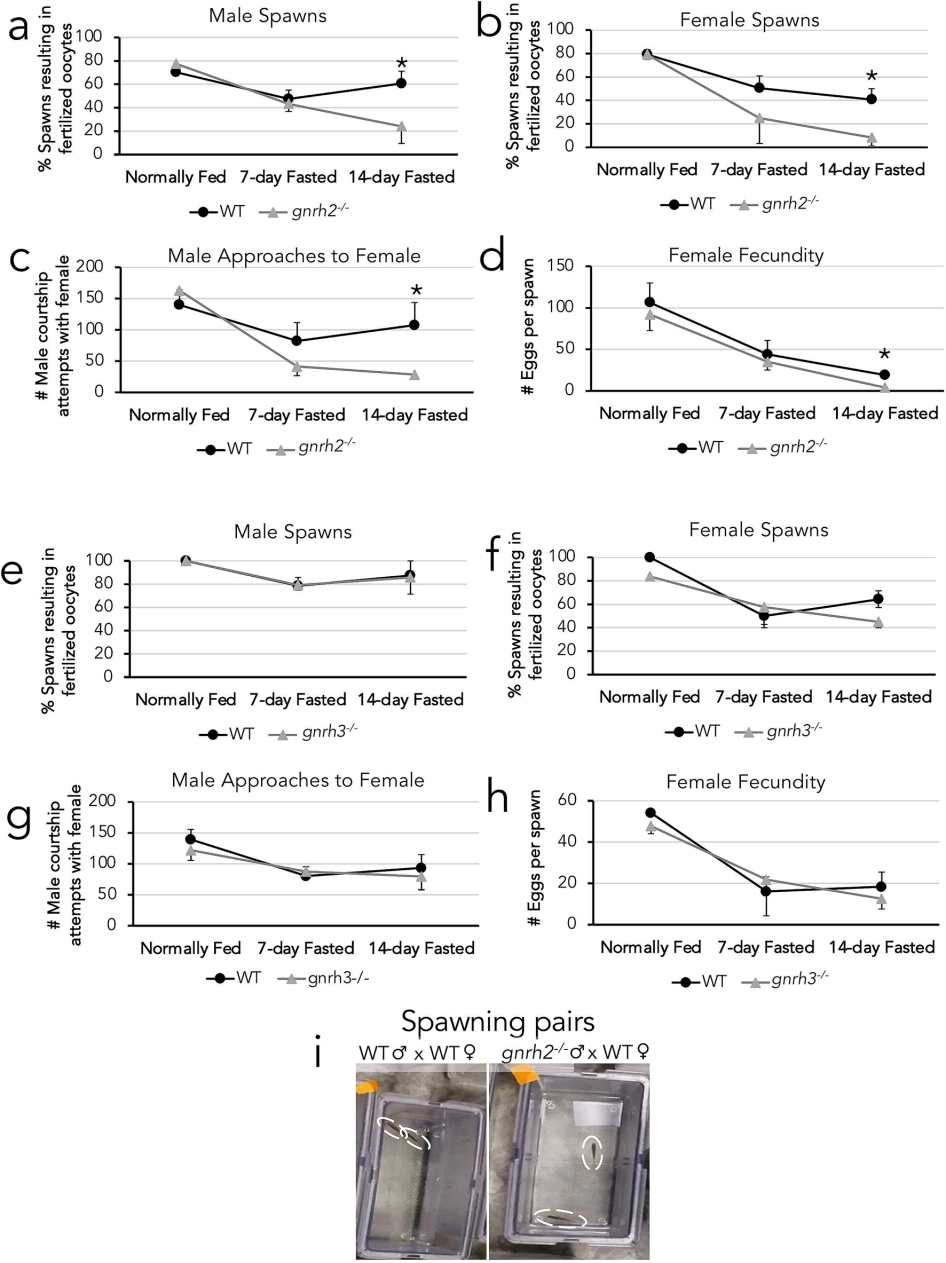
Reproductive outputs of male (n = 8–10 per group) and female (n = 5–8 per group) WT, gnrh2, and gnrh3 fish undergoing 0, 7, and 14-day fasting, including WT and *gnrh2*^−/−^ comparisons of male spawning percentages (a), female spawning percentages (b), quantification of male courtship attempts with female partners (c), and female fecundities (d). WT and *gnrh3*^−/−^ comparisons of male spawning percentages (e), female spawning percentages (f), quantification of male courtship attempts with female partners (g), and female fecundities (h). Still image from video of spawning tanks demonstrating typical behavior of WT males chasing females and *gnrh2*^−/−^ males less frequently chasing female partners (i) All data expressed as means ± S.E.M, *P < 0.05.

Finally, we compared reproductive outputs between *gnrh2* mutants and their WT counterparts, as well as *gnrh3* mutants and their WT counterparts (n = 8–10 males and 5–8 females/group). WT and *gnrh2*^−/−^ males (Fig. 6a) and females (Fig. 6b) exhibited similar rates of spawning at normally fed and 7-day fasting conditions but exhibited decreased spawning rates at 14 days of fasting compared to their WT counterparts (P < 0.05), with only 8% of *gnrh2*^−/−^ females spawning compared to 40% WT females (Fig. 6b). *Gnrh2*^−/−^ males also initiated courtship attempts with females significantly less (about three-fold difference) than WT males at 14 days of fasting (Fig. 6c, i) (P < 0.05) but showed similar courtship rates at 0 and 7 days fasting (Fig. 6c). *Gnrh2*^−/−^ females had similar fecundity numbers at 0 and 7 days fasting but had significantly decreased fecundity than WT counterparts at 14 days fasting (Fig. 6d) (P < 0.05). *Gnrh3* mutant males (Fig. 6e) and females (Fig. 6f) exhibited similar spawning rates to their WT counterparts for all feeding conditions tested. Similarly, both male courtship attempts with females (Fig. 6g) and female fecundity rates (Fig. 6h) were not significantly different between *gnrh3*^−/−^ and WT zebrafish.

## Discussion

In this study, we analyzed the role of Gnrh2, a neuropeptide that was previously found to both reduce feeding behaviors and stimulate reproductive behaviors^24,28,30^, in gonadotropin synthesis and reproduction maintenance under fasting conditions using the zebrafish model. We observed that Gnrh2 becomes a critical regulator that upholds reproduction in zebrafish during fasting. Gnrh2 neurons and protein increased in abundance in the pituitary after 14 days of fasting, and its presence was important in maintaining high levels of gonadotropin expression, oocyte maturation, and subsequent oviposition after a 14-day fasting period.

We first examined Gnrh2 and Gnrh3 neuronal pituitary patterns in fed, 7-day fasted, and 14-day fasted conditions. In zebrafish, one week of fasting induces changes in both brain and gut appetite-regulating genes^35,36^ and two weeks of fasting induces liver protein and other enzyme responses^37^ and previously shown to induce plasticity of Gnrh2^33^. Therefore, we chose two weeks fasting as our end point in this analysis, of which there was no mortality in any of our experiments. We found that Gnrh3 neuronal innervations to the pituitary decrease, whereas that of Gnrh2 increase in two-week fasted zebrafish. In zebrafish, Gnrh1 was evolutionarily lost, but Gnrh3, which is present in the same reproductive preoptic area, is considered to function similarly to the hypophysiotropic Gnrh1 as a central reproductive regulator. Recently, however, the importance of Gnrh3 as a major reproductive regulator in zebrafish has been brought into question. On one hand, genetic knockouts of Gnrh3 in zebrafish surprisingly result in no known negative effects on reproductive performance^38–40^ except for reduced PGC numbers and a male-biased sex ratio^41^. This raised the possibility of a compensatory response by other genes^42–44^, which was indicated by upregulation of other neuropeptide genes including *scg2a*, *pacap1*, and *tac3a*, but not *gnrh2*, in *gnrh2*^−/−^; *gnrh3*^−/−^^40^ and *kiss1*^−/−^*;kiss2*^−/−^*;gnrh3*^−/−^ mutants^41^. On the other hand, targeted ablation of Gnrh3 neurons in zebrafish results in failed oocyte development and female infertility, implying a critical role of Gnrh3 neurons in reproduction and gametogenesis in this species^45^. Regardless of the exact role of Gnrh3 in reproduction, overall brain transcript levels of *gnrh3* was slightly reduced after two weeks of food deprivation in zebrafish, with a more dramatic reduction in the actual presence of the neurons and subsequent Gnrh3 protein in pituitaries. Supportingly, previous studies in other fish species show downregulation of the hypothalamic Gnrh after fasting, including in glass catfish and *Astatotilapia burtoni*, where food deprivation induced downregulation of Gnrh1 without negatively affecting Gnrh2^22,46^.

In contrast, Gnrh2 neurons showed a different pattern and fasted zebrafish displayed increased innervations and Gnrh2 protein content in the pituitary. Both antibody stains and ELISA assays confirmed the increase of Gnrh2 protein abundance after a 14-day fasting period. Importantly, the increased presence of Gnrh2 neurons in the pituitary are not induced in *gnrh3*^−/−^ zebrafish, indicating that feeding cues, instead of the loss of Gnrh3, are directing this change. The presence and abundance of Gnrh2 in the pituitary of other teleosts appears to be species-specific, with some species demonstrating undetectable Gnrh2 presence^47^, others exhibiting low Gnrh2 presence^48^, and some containing abundant Gnrh2 presence in the pituitary^49^. Considering the dynamic plasticity of Gnrh neurons, it is possible that Gnrh2 presence may differ in these species if they are examined in different feeding conditions, such as in the case of the musk shrew. After food restriction, Gnrh2 neuronal soma in the midbrain and fiber density in the median eminence of female musk shrews increased by 50% compared to fed conditions^32^. This same response was demonstrated in zebrafish, with an abundant fasting-induced increase of Gnrh2 neuronal projections to the pituitary^33^, suggesting a conserved response in several species. It remains to be answered what drives the same response of Gnrh2 neurons to food restriction in species like zebrafish and musk shrew.

Next, we examined whether the increased Gnrh2 neuron terminals reach gonadotropin cells and concluded they increase in proximity to both Lh and Fsh gonadotropin cells after fasting. The Gnrh2 neurons appear to localize more abundantly to the proximal pars distalis (PPD), where the gonadotrope cells are located, with some localizations to the rostral pars distalis (RPD) as well. Future studies will need to determine whether Gnrh2 neurons are terminating on and stimulating other types of pituitary cells in the RPD as well. Gnrh2 has been shown to stimulate Lh in fed zebrafish^28^ and Lh and Fsh transcription and protein secretion in goldfish^50^, gilthead seabream^51^, striped bass^52^, and catfish^53^. Moreover, several studies show Gnrh2 having equal or higher potency than the major hypophysiotropic Gnrh’s (Gnrh1 in three-Gnrh species and Gnrh3 in two-Gnrh teleost species) at eliciting gonadotropin secretion from the pituitary of teleosts such as goldfish, catfish, and seabream^51–55^. Hence, Gnrh2 is capable of potently activating Gnrh receptors on gonadotropes of many teleosts and may contribute to stimulating gonadotropin synthesis in fasted zebrafish as well. Supportingly, zebrafish Gnrh2 has previously been shown to bind and activate the inositol phosphate (IP) signaling pathway with all four native Gnrh receptors and with equal potency as Gnrh3 with GnrhR2 and GnrhR4 and a much higher potency with GnrhR1 and GnrhR3^56^. In our study, we detected notable expression levels of *gnrhr1*, *gnrhr3*, and *gnrhr4*, but not *gnrhr2*. It is possible that *gnrhr2* mRNA is present but is under the detected limits of our assay.

Supporting the idea that Gnrh2 helps to maintain gonadotropin synthesis in fasted conditions, only WT fish, but not *gnrh2*^−/−^*,* had levels of Lh and Fsh mRNA and protein similar to those of normally fed fish after 14 days of fasting, suggesting the presence of Gnrh2 in the pituitary is critical to stimulate gonadotropin expression in fasted states. WT fish displayed decreased gonadotropin expression and reproductive outputs at 7 days fasting, but increased gonadotropin levels and reproduction at 14 days of fasting, while in *gnrh2*^−/−^ fish, there was no recovery of gonadotropins or reproduction at 14-days fasting. Since WT Gnrh2 neuronal projections also increase their proximity to gonadotropins at this time, we propose that Gnrh2 is responsible for inducing this increase in gonadotropin levels. After two weeks of fasting, transcript levels of *gnrh2* and *gnrh3* in brain tissues showed no significant changes; however, there was a significant increase in Gnrh2 protein content in the pituitaries of fasted zebrafish, signifying this was a result of the increased Gnrh2 neuron terminals in the pituitary and not an increase in total expression after fasting. Fasted males and females showed no differences in the expression of Gnrh receptors in the pituitary, suggesting that there is most likely no difference in the sensitivity of gonadotropin cells to Gnrh ligands after fasting, and therefore, the increased gonadotropin release is a consequence of increased Gnrh2 protein due to the higher prevalence of Gnrh2 neuron terminals.

We also analyzed the Gnrh2 loss-of-function consequences on gametogenesis after fasting. Zebrafish are asynchronous daily batch spawners and have a mix of oogonia and oocytes at different stages of maturation, with previous studies showing that during development, it takes around two weeks for a primary-growth follicle to develop into a fully mature oocyte^57^. The loss of Gnrh2 in normally fed zebrafish was previously shown to be associated with decreased GSI and slightly decreased oocyte quality^28^. Here, we observed that in normally fed conditions, *gnrh2*^−/−^ female zebrafish also exhibited decreased GSI but maintained the ability to produce mature oocytes. After a two-week fasting period, *gnrh2*^−/−^ zebrafish exhibited an extreme reduction in mature oocyte production, whereas wild-type fish contained significantly increased percentages of vitellogenic and mature oocytes. This suggests Gnrh2 plays a minor role in gonadal maturation and growth in normally fed conditions but is a more critical regulator of oocyte maturation after fasting, which likely occurs through its upstream regulation of Lh. Similar to mammals, previous studies in zebrafish and medaka show that Lh is particularly important for final oocyte maturation^58,59^. It is possible that the fasted *gnrh2*^−/−^ females did not have the adequate levels of Lh to maintain late oocyte maturation and oocytes instead stayed stalled at the oogonia/primary growth/cortical alveoli stages and, to a lesser extent, vitellogenic stages. *Gnrh2*^−/−^ fasted ovaries showed the presence of late atretic follicles, suggesting some oocytes at the late maturing stage may have underwent atresia. Ovaries from fasted WT zebrafish also showed indications of some late atretic oocytes, as well as oocytes beginning to undergo early atresia. Further analysis on steroid production and apoptosis in these animals will be critical to identify the exact nature of gametogenic differences between the knockout and wild-type zebrafish. The loss of Gnrh2 did not, however, result in any differences in spermatogenesis. Several studies demonstrated that Lh is not as important for regulating spermatogenesis^58^, indicating the reduction in Lh in *gnrh2*^−/−^ fish may more prominently affect gametogenesis in females. The differential regulation of male and female gametogenesis by Lh most likely explains the observation that the loss of Gnrh2 did not result in any differences in spermatogenesis. These results, combined with the fact that Gnrh2 protein levels demonstrated 3 to fivefold higher levels in female pituitaries compared to males, indicate that Gnrh2 may be a more significant regulator of gametogenesis in females. Supportingly, we have previously discovered the loss of Gnrh2 in zebrafish results in some perturbations to oocyte quality, but not sperm quality^28^. Further studies on the upstream regulation of the Gnrh ligands and gonadotropins on steroidogenesis and gametogenesis in zebrafish along with further analysis on apoptosis levels in the gametes will help obtain a clearer idea of reproductive control at the gonadal level.

Finally, we analyzed the role of Gnrh2 in reproductive behaviors and outputs after fasting through examining the percentage of successful spawns resulting in fertilized oocyte production, female fecundity, and male courtship behaviors. We observed *gnrh2*^−/−^ females and males to exhibit significantly less successful spawns after 14 days of fasting, whereas WT and Gnrh2 mutant zebrafish undergoing normally fed and 7-day fasting conditions resulted in no differences in reproductive outputs, implying that Gnrh2 may be a more critical regulator of reproduction after 14-day fasting. Additionally, *gnrh3*^−/−^ fish were able to successfully spawn, with no major male behavior or fecundity differences, after 14 days of fasting, inferring that the sole presence of Gnrh2 is enough to maintain reproduction in fasted states. Interestingly, although male spermatozoa production was normal in *gnrh2*^−/−^ fish, fasted males were still not eliciting successful spawns from fed WT females, which may have been due to the decrease in male courtship behaviors displayed by these same males. Gonadotropins are known to stimulate steroidogenesis^60^ and steroids are critical to evoke rapid male reproductive behaviors during mating^61^. Therefore, the reduced male reproductive behaviors seen in fasted *gnrh2*^−/−^ males may be an indirect consequence of reduced steroidogenic activities. Another possibility is that Gnrh2 projections to olfactory bulb regions, as previously described^33^, help to transduce steroid olfactory signals to influence male behaviors, which is lost in the mutant line.

Our results overall indicate key roles of Gnrh2 in centrally regulating reproduction downstream of anorexic cues in zebrafish. In mammals and fish, Gnrh2 was found to both stimulate reproduction and decrease feeding behaviors^26,28,32^ through mediating and responding to feeding peptides, supporting this hypothesis. Since fasting periods are frequently associated with the reproductive season in teleosts, as well as other vertebrates, knowledge on the neuroendocrine control of reproduction in this condition is important to understand environmentally relevant situations. Previous research on the response of Gnrh2 to fasting conditions indicate species-specific expression changes, however. For instance, during fasting periods, winter flounder exhibited decreased *gnrh2* and *gnrh3* mRNA ^62^, but other species such as Atlantic cod^63^, glass catfish^22^, and African cichlid^46^ showed no change in *gnrh2* mRNA levels after fasting^63^. These variations may be due to different experimental measurements and methodologies, variations in feeding and reproductive strategies, or indicate species-specific variations in the reproductive roles of Gnrh peptides in fasting states. Overall, this suggests that expression levels may not be an adequate sole indicator for reproductive adaption to fasting. Our study is one of the first to examine both transcript and peptide Gnrh abundances, which show different responses, indicating that a thorough look at both transcription and translation may be needed for a comprehensive understanding of Gnrh response to feeding and fasting. In our study, although Gnrh transcript levels did not exhibit significant changes, there were still significant increases in pituitary Gnrh2 protein levels and neuronal projection localization, suggesting that neuronal projection patterning changes instead of mRNA-associated expression changes may be directing the Gnrh response to fasting. Thus, although we show the importance of Gnrh2 in reproduction during fasting in zebrafish, studies on Gnrh2 in other species will need to be conducted to determine the extent of the conserved roles of Gnrh2 in other vertebrates.

Overall, our results suggest that Gnrh2 may be a major stimulator of gonadotropin synthesis and subsequent reproductive performance in fasted zebrafish. Further research will need to be conducted in other species to determine the extent of the conserved roles of Gnrh2, which may be of particular interest in teleosts which have a conserved Gnrh2 system and the ability to reproduce after extremely long periods of fasting, such as in the migratory and important aquaculture species of salmon and eel^14^. Here, we show that the increased role of Gnrh2 as a key reproductive regulator in fasted states is mediating by gonadotropin synthesis and associated downstream targets.

## Methods

### Fish maintenance

Zebrafish were raised and maintained in the in-house zebrafish facility at the Institute of Marine and Environmental Technology (IMET). Adult zebrafish were kept in a recirculating water system at 28 °C with a 14L:10D photoperiod cycle, with water quality parameters checked daily by zebrafish husbandry staff. Zebrafish embryos/larvae from 5 to 14 days post fertilization (dpf) were kept on a nursery rack and fed ad libitum with *Paramecium*, and zebrafish larvae from 14 to 30 dpf were fed ad libitum with *Artemia*. After 30 dpf, zebrafish were moved to the recirculating system and fed, twice daily, with 300–500 µM Gemma pellets (Skretting). Mutant and WT lines used in the analysis were from the Tübingen background. All zebrafish of the same genotype were either siblings or cousins of each other (spawned over three months from the same parent cohort) and wild-type and knockout counterparts which were compared in analyses were cousins with shared grandparents to reduce variation. Sexual maturation of zebrafish from this background was confirmed to occur at 2–3 months of age. Zebrafish sampling occurred via euthanasia with 300 mg/L Tricaine (MS-222, Sigma-Aldrich) and subsequent decapitation. All zebrafish used in the experiments were 6–8 months of age, confirmed to successfully spawn, and then moved to 10-gallon (37.85 L) tanks which reside in the main recirculating system of our zebrafish facility, and fed normally for two weeks (approximately 3% of BW with 300–500 µL Gemma pellets twice daily) before the beginning of the experiments. Any fish which were spawned for reproductive assessments were moved to a 2L breeder the night before spawning, paired with a fed WT partner, and separated by a divider. The morning of spawning, the divider was removed as soon as lights were turned on and fish allowed to spawn for one h. Three different feeding conditions used in the experiments consisted of a normal feeding (approximately 3% of BW of 300–500 µL Gemma pellets twice daily), 7-day fasting (normal feeding for one week followed by complete withholding of food for 7 days), or 14-day fasting (complete withholding of food for 14 days). The time period of fasting has been frequently used in other zebrafish feeding assays and shown to induce transcriptional and hormonal changes^32–34^ but did not result in any mortality of fish (in our experiments). Specific samples sizes, sex proportions, and protocols for each experiment are described below. All zebrafish protocols were approved by the Institutional Animal Care and Use Committee (IACUC) at the University of Maryland School of Medicine and all experiments were performed in accordance with IACUC approved guidelines and regulations. Additionally, all experiments in this study were carried out in compliance with the ARRIVE guidelines.

### Confocal microscopy characterization of transgenic zebrafish pituitaries

In order to characterize Gnrh2 and Gnrh3 neuronal projections and examine Gnrh2 neuronal innervations with Lh cells, pituitaries from *tg(gnrh2:eGFP)*^33^, *tg(gnrh3:tdTomato)*^33,64^, or *tg(gnrh2:eGFP;lh:mCherry)*^65^ zebrafish were dissected and transferred to an artificial cerebrospinal fluid (ACSF: 119 mM NaCl, 26.2 mM NaHCO3, 2.5 mM KCl, 1 mM NaH2PO4, 1.3 mM MgCl2, 10 mM glucose, 2.5 mM CaCl2, plus 95% O2/5% CO2) bath. Zebrafish used in the experiment were 6 months old, confirmed to successfully spawn, and kept in two separate 10-gallon tanks in the main recirculating system. One tank, consisting of six males and six females, was normally fed for 14 days, whereas the other tank was fasted for the same time period. Small mesh was placed on the intake water supply, to allow water flow but prevent any accidental food sources from getting into the tanks. At the end of two weeks, all zebrafish were euthanized and pituitaries quickly dissected (n = 6 per group) and placed in the ACSF solution. For imaging pituitaries, a small piece of coverslip was gently placed on top of pituitaries to flatten them for imaging. Images were taken with a Leica TCS SP8 confocal microscope at 5X and 20X magnification and using the Leica Application Suite X (https://www.leica-microsystems.com/products/microscope-software/p/leica-las-x-ls/). Z-stack projections and neuronal fiber length measurements were conducted using Fiji^66^. Additionally, for double transgenic *tg(Gnrh2:eGFP; Lh:mCherry)* pituitaries, a confocal z-stack image was taken using the Leica TCS SP8 microscope (at 5X magnification) and a max projection of the image was produced using Fiji, which allowed the visualization of the entire pituitary tissue. Using the max projection image, the total amount of visible Lh cells next to Gnrh2 fibers were counted using the manual cell counter plugin^67^ of Fiji for each pituitary. Six pituitaries were analyzed and quantified, and the overall number of Lh cells next to Gnrh2 fibers averaged.

### Immunohistochemistry of Gnrh2, Gnrh3, and Fsh in zebrafish pituitaries

The zebrafish used for the immunohistochemistry experiments consisted of WT siblings as those used in the spawning experiments (with shared grandparents of Gnrh2 knockout counterparts). Six males and females (7 months in age) for each feeding condition (a total of 12 males and 12 males in total) were confirmed to spawn successfully, moved to two separate 10-gallon (37.85 L) tanks and fed normally for two weeks. Then, while one tank was normally fed (approximately 3% of BW twice daily), the other tank was fasted for the duration of two weeks. At the end of two weeks, zebrafish were euthanized and pituitaries quickly dissected and placed in 4% PFA for overnight fixation. The next day, the pituitaries were immersed in 30% sucrose in PBS until the tissue sunk to the bottom of the tube. Samples were then mounted and frozen in OCT, sectioned to 10 µM thickness using a Tissue-Tek Cryo3 cryostat, and mounted on Plus coated slides. Immunohistochemistry was performed to verify the Gnrh2 and Gnrh3 neuronal protein abundance in pituitaries and to analyze Gnrh2 and Fsh interactions in the pituitary. Antibodies used were previously validated and generated against the specific recombinant zebrafish GAP region of Gnrh2 (GAP2)^33^, the specific recombinant GAP region of Gnrh3 (GAP3)^38^, and against the beta subunit of carp Fsh, which shares a highly conserved sequence identity with zebrafish^68^. To perform IHC, slides were briefly fixed in acetone and quenched in 0.3% H_2_O_2_ in PBS for 30 min to remove endogenous peroxidases. Slides were then washed in PBS, blocked for 1 h in 5% normal goat serum, and incubated with primary antibody (Anti-GAP2 or Anti-GAP3, 1:1,000) in 1% BSA and 0.3% Triton X-100 overnight at 4 °C. Slides were washed in TNT (100 mM Tris Ph 7.5, 150 mM NaCl, 0.5% Tween-20) and incubated in an HRP-conjugated Goat anti-Rabbit (GAR-HRP) antibody (Genscript) at a 1:1,000 dilution in 1% BSA and 0.3% Triton for 1 h. Then, slides were washed in TNT and incubated in a fluorescein dye from the Tyramide Signal Amplification Plus kit (TSA Plus kit, Perkin Elmer) at a 1:50 dilution for 5 min, washed in TNT, and HRP signal quenched with 0.02 N HCl for 10 min if being used for double-labelling. For double-labelled slides, after washing, the procedure for IHC delineated above was repeated with a carp-Fsh primary antibody (1:200) and Cy3 dye (1:50) from the TSA kit to label FSH. Slides were mounted in 50% glycerol plus 10 µg/ml Hoescht 33,342 (Sigma) and imaged at 5X and 20X magnification using a Leica TCS SP8 confocal microscope. Images were compiled and quantified using Fiji^66^. For each measurement, six pituitaries were sectioned and five sections from each pituitary, encompassing the entirety of the tissue, was quantified by counting either the length of Gnrh2 or Gnrh3 projections or the number of Fsh cells in contact with Gnrh2, using the cell counter plugin in Fiji^67^.

### qPCR Analysis of Gnrh ligands, receptors, and gonadotropins

In order to determine fasting-induced expression differences of Gnrh ligands, receptors, and gonadotropins, WT and Gnrh2 knockout counterparts (7–8 months of age) were confirmed to successfully spawn, moved to 10-gallon tanks, and normally fed for two weeks before the start of the experimental feeding conditions. WT and Gnrh2 knockout fish were each placed in two separate tanks (four tanks total), each tank consisting of 8 fish from each sex. One tank of each genotype was normally fed, whereas the other tank was fasted for 14 days. For gonadotropin expression analysis, an additional tank was normally fed for one week and fasted for 7 days. On day 14, all zebrafish were euthanized and brain and pituitary tissues dissected from each fish and frozen in dry ice and stored at -80 °C. Brain tissues were used to analyze differences in the gene expression levels of *gnrh2* and *gnrh3* and pituitary tissues were used to analyze differences in *gnrhr1, gnrhr3, gnrhr4, lhb,* and *fshb* expression^69^. Brain and pituitary tissues were homogenized through sonification in DEPC-treated sterile milliQ water using a Sonifier 450 (Branson, Inc.) with duty cycle settings at 100% and output control at 3. For pituitaries, 100 µL of Trizol reagent was added, and for brains, 300 µL was added to each sample. Total RNA was extracted from each sample according to the standard manufacturer’s protocol. Reverse transcription was conducted using the Quantitect Reverse Transcription Kit (Qiagen) along with gDNA Wipeout to obtain a total of 1 µg cDNA for each sample. For each reverse transcription experiment, a sample with no reverse transcriptase enzyme added was used as a non-RT control. For qPCR analyses, 20 ng of cDNA for each sample was used, in duplicate, with SYBR Green qPCR mix and specific primers (Table 1) and the qPCR ran on a 7500 Fast Real-Time PCR System (Thermo Fisher Scientific). The protocol for amplification included a 95 °C activation for 2 min, 95 °C denaturation for 5 s, and 60 °C annealing for 30 s, with 40 repeating cycles. Relative expression values were quantified using the 2^-(∆∆CT)^ method. All expression values were normalized to an internal *eef1a1* housekeeping gene, which has frequently been validated as one of the most stable housekeeping genes between different zebrafish adult tissues^70–72^ and found to show the most stable expression between zebrafish brain tissues^73^.

**Table 1:** Sequences of forward and reverse primers used in the qPCR reactions.

Primers	Sequences (5′ to 3′)
Eef1a1	Fw: AAGACAACCCCAAGGCTCTCA
	Rv: CCTTTGGAACGGTGTGATTGA
Fshb	Fw: GCTGGACAATGGATCGAGTTTA
	Rv: CTCGTAGCTCTTGTACATCAAGTT
Gnrh2	Fw: CAGAGGTTTCAGAGGAAGTGAAGC
	Rv: TGAGGGCATCCAGCAGTATTG
Gnrh3	Fw: TGGAGGCAACATTCAGGATGT
	Rv: CCACCTCATTCACTATGTGTATTGG
Gnrhr1	Fw: CTGCTTCAGTAGGAGGAATCAA
	Rv: CCAAGGTCTGACTCTGCTTC
Gnrhr3	Fw: CTGCGACCCTGTGATCTATG
	Rv: TATGTGTGTCTGGTGGGTTG
Gnrhr4	Fw: CTCATTGAAATGCTCACCATCC
	Rv: CGTCAGCTGCCTTCTTATCT
Lhb	Fw: GGCTGGAAATGGTGTCTTCT
	Rv: CCACCGATACCGTCTCATTTAC

### Lh and Fsh ELISA

In order to determine the effect of fasting on Lh and Fsh protein production between WT and Gnrh2 knockout zebrafish, pituitaries were dissected from normally fed, 7-day fasted, and 14-day fasted fish. WT and Gnrh2 knockout counterparts (7 months of age) were confirmed to successfully spawn, and then moved to 10-gallon tanks and normally fed for two weeks before the start of the experimental feeding conditions. WT and Gnrh2 knockout fish were each placed in three separate tanks (six tanks total), each tank consisting of 8 fish from each sex. One tank of each genotype was normally fed, one was normally fed for 7 days and then fasted for 7 days, and the other tank was fasted for 14 days. On the morning of the 14^th^ day, each fish was quickly euthanized and tissues dissected. For gonadotropin protein extraction, pituitary tissues were dissected from adult zebrafish, individually placed in a 1.5 mL tube, and quickly placed on ice. For pituitary homogenization before the ELISA assay, 200 µL of PBST (0.05% Tween 20 in PBS) solution was added to the pituitary tissue, and pituitaries were homogenized via sonication for 20 s each (Sonifier 450, duty cycle 100%, and output cycle at 3). 200 µL of the homogenized pituitary solution was added to 200 µL of 4 N acetic acid, for a final concentration of 2 N acetic acid. Tubes were immediately vortexed and placed on ice for 15 min. Samples were centrifuged for 30 min at 14,000 RPM, and the supernatants collected and frozen at −80 °C until the ELISA was conducted. The Lh and Fsh ELISA protocol was performed using carp recombinant beta proteins which were developed and optimized for use in zebrafish gonadotropin quantification previously^68^, with minor modifications for our use. Just prior to the ELISA assay, samples were lyophilized overnight, reconstituted in 250 µL of sterile milliQ water, briefly centrifuged, and 125 µL supernatant transferred to 2 new tubes (one for Fsh ELISA, one for Lh ELISA). For the ELISA, 96-well ELISA plates were coated with either 100 µL of beta-Fsh in 50 mM NaHCO3 (pH 9.6) (final concentration of 2.5 ng/mL of beta-Fsh) or 100 µL of beta-LH in 50 mM NaHCO3 (pH 9.6) (final concentration of 20 ng/mL of beta-Lh). Standards were set up by diluting beta-alpha Fsh to 20 ng/ml in 250 uL assay buffer (PBS, with 0.05% Tween 20 and 0.01% BSA) with 10 subsequent two-fold serial dilutions (11 tubes total, ranging from 20 ng/mL to 9.7 pg/mL) or beta-alpha Lh to 210 ng/ml in 250 uL assay buffer with 10 subsequent three-fold serial dilutions (11 tubes total, ranging from 210 ng/mL to 1.2 pg/mL). The sensitivity of the assays (lower limits of detection) for Lh and Fsh concentration were previously determined to be 32 pg/mL and 7 pg/mL, respectively^68^. After standards and samples were set up, 125 uL of Fsh or Lh antibody (1:1,000 in assay buffer), in duplicates, were added to each standard or sample tube. All tubes and coated plates were covered and incubated overnight at RT. The next day, coated plates were washed 3X in PBST at 200 µL/well. 200 µL of blocking buffer (PBST plus 1% BSA) was added to each well, and plates then covered and incubated for 30 min at 37 °C. Plates were then washed 3X, 100 µL of standard or sample was added to each well, and plates incubated at 37 °C for 1.5 h and then washed 3X. Secondary antibody consisting of 100 uL of GAR-HRP (1:5,000 in PBS-T 0.1% BSA) was added to each well and plates incubated at 37 °C for 1 h. Plates were washed 3X and then 100 µL of 1:2 diluted 3,3′,5,5′-Tetramethylbenzidine (TMB ELISA Substrate, Abcam) added to each well. Plates were covered and placed in the dark for approximately 1 h (for Lh) or 20 min (For Fsh), and then the reaction stopped with 100 µL of 1 N phosphoric acid. OD readings were subsequently conducted using a spectrophotometer at 450 nm. The inter-assay coefficient of variability (CV) for the Lh and Fsh ELISA assays were previously determined to be 11.3% and 8.66% respectively^68^.

### Gnrh2 ELISA

In order to determine the effect of fasting on Gnrh2 protein presence in WT and Gnrh2 knockout zebrafish pituitaries, groups of WT and Gnrh2 knockout fish (8 fish of each sex per feeding condition which were siblings to the fish used in the Lh/Fsh ELISA experiment) were preliminarily tested for spawning, moved to a 10 gallon tank, and normally fed for two weeks prior to experimentation, and then either normally fed or fasted for 14 days in the same manner as described above (Lh/Fsh ELISA). On the 14^th^ day, fish were quickly euthanized and pituitaries dissected and placed on ice. Directly after pituitary dissection, the gonads were also carefully dissected (see gonad histology section for further details). Pituitary tissues were then homogenized and extracted according to the same procedures above (LH and FSH ELISA protocol) and stored at -80 °C until ready for the ELISA. Gnrh2 ELISA was conducted as described previously, with minor modifications for our use^74^. Just prior to the ELISA assay, samples were lyophilized overnight, reconstituted in 250 µL of sterile milliQ water, briefly centrifuged, and supernatant collected. For the ELISA, 96-well plates were coated with goat anti-rabbit IgG (200 µL/well) overnight at a concentration of 10 µg/mL in potassium phosphate buffer. Blocking buffer (0.3% BSA, 0.9% NaCl, 1.8% K2HPO4, 3% KH2PO4, 1 mM EDTA, 5 mM sodium azide) was then added at a volume of 200 µL/well, and plates stored for a minimum of 4 h at RT. Plates were washed 3X in PBST, and 50 µL of samples or standards, in duplicates, added to each well. Standards consisted of Gnrh2 peptide diluted to known concentrations of 10 ng/mL to 20 pg/mL with the lowest detectable limit of the assay previously determined to by 7 pg/well^74^. Lyophilized Gnrh2-aCHE (Acetylcholine Estherase) tracer (produced by Cayman Chemicals Inc.) was reconstituted to 8 ng/mL in 5 mL of assay buffer (0.9% NaCl, 1.8% K2HPO4, 3% KH2PO4, 0.1% BSA, 0.15 mM sodium azide) and 50 µL added to all wells but one (NSB- non-specific binding) control well. Specific Gnrh2 anti-decapeptide antibody, (kindly provided by the late Dr. Judy King), was diluted to a final concentration of 1:15,000 in assay buffer and 50 uL added to each well but one control well. Plates were covered and incubated for 3 days at 4 °C. ELISA plates were then washed 3X with 200 µL/well of PBST, and then 200 µL of Ellmann’s reagent (0.9% NaCl, 1.8% K2HPO4, 3% KH2PO4, 215 mg DNTB (5,5′-Dithiobis(2-nitro-benzoic acid)) (sigma-D8120), 200 mg acetyl thiocholine) was added to each well. Plates were covered and placed in a dark location for approximately 3 h, or until the color developed to 1.5–2.0 OD. OD readings were subsequently quantified using a spectrophotometer at 405 nm.

### Gonad histology and GSI characterization

To assess gonad morphology of wild-type and knockout zebrafish in fed and fasted conditions, ovaries and testes from normally fed or 14-day fasted mature zebrafish were dissected from the same animals of which the pituitaries were harvested in the Gnrh2 ELISA assay (see above details in Gnrh2 ELISA section for zebrafish tank and feeding experimental conditions). Each fish (n = 8 for each sex, genotype, and feeding condition) was quickly euthanized on the same morning, weighed to obtain total body weights, and then gonads were carefully dissected, weighed, and fixed in 4% PFA overnight. Gonad tissues were then paraffin-embedded, cut into 10 µm thick sections, rehydrated, and stained with hematoxylin and eosin according to the manufacturer’s protocol (Sigma-Aldrich, St. Louis, MO, USA). Ovarian and testicular sections were imaged using the bright field setting of a Zeiss Axioplan2 microscope and CCD Olympus DP70 camera with a scale bar. Oocytes were classified into three different maturational stages, based on the five stages of maturation classified previously and frequently used in zebrafish histological classification^75,76^. Oocytes at stages 1 and 2 (oogonia, primary growth, and cortical alveolus stages) were classified as “pre-vitellogenic” by their diameters under 250 mm and the absence of yolk granules. Oocytes at stage 3 (vitellogenin uptake stage) were classified as “early-mid vitellogenic” by their diameters between 250 and 500 mm, presence of vitellogenin/yolk granules, and nuclear membrane. Oocytes at stages 4 and 5 (late oocyte maturation and fully mature stage) were classified as “late/fully mature” by the presence of germinal vesicle migration or breakdown (GVBD) respectively and diameters greater than 500 mm. The total number of oocytes at each stage was counted for each section (eight different sections were counted for each of the eight ovaries, representing the entire width of the tissue), and then summed, averaged, and quantified as a proportion of the total oocyte population for each ovary. Before fixation of gonad tissues, gonadosomatic index (GSI) was quantified for each individual as the percentage of gonadal to somatic weight using the formula ([gonad weight / somatic weight] X 100).

### Reproductive assessments

In order to analyze the effects of the loss of Gnrh2 and Gnrh3 on fasting-induced reproductive outputs, *gnrh2*^−/−^, *gnrh3*^−/−^, and WT zebrafish (7–8 months of age) were exposed to three different feeding regimes consisting of normal feeding (3% BW of 300–500 µL Gemma pellets), 7-day fasting (one week of normal feeding followed by one week of no food), and 14-day fasting (two weeks of no food). All zebrafish were first confirmed to spawn successfully, moved to a 10-gallon tank, and normally fed for two weeks. Zebrafish which were compared between the genotypes were preliminarily matched by age (7–8 months old) and similar sizes in order to reduce physiological variability. All *gnrh2*^−/−^ and *gnrh3*^−/−^ fish used in the experiment were compared to related WT counterparts and siblings or cousins to the zebrafish used in the previous experiments. Six tanks were set up, with one tank of females (n = 5–8) and one tank of males (n = 8–10) set up for each genotype. A male Casper fish was placed in each female tank and a female Casper fish was placed in each male tank to ensure optimal reproductive health of all experimental zebrafish. First, all fish were normally fed and the evening before spawning was being analyzed, they were placed in a separate 2L breeding tank, paired with a fed WT partner (to reduce partner variability), and separated by a divider. The following morning, once the light was turned on, dividers were removed and fish allowed to spawn for one h. The presence of fertilized eggs after one h of spawning was an indicator of a successful spawn. In order to determine fecundity, the total number of oviposited eggs was counted for each female. After the one h spawning period was finished, fish which successfully spawned were placed back in tanks and fasted for 7 and 14 days and the same experimental protocol repeated. This experiment was repeated three separate times with different groups of fish.

### Male reproductive behavior

To determine male reproductive behaviors, mature male fish which were used in the reproductive assessment experiments (detailed above) were additionally filmed in order to determine reproductive behaviors. The following morning after spawning tanks were set up, dividers were removed as soon as the light was turned on and tanks containing experimental males were filmed with a GoPro Hero 3 camera for a 10 min period of time. Tanks were intermixed and marked with a label to identify genotype. The number of times males approached females and initiated courtship behaviors was counted for each tank, and tanks were retrospectively matched to the correct genotype to reduce bias.

### Statistical Analysis

All statistical analyses were conducted using R statistical software version 3.6 (https://www.r-project.org/). One-way ANOVA with subsequent Tukey’s post-hoc test for multiple comparisons was used to analyze significant differences in pituitary fiber length projection measurements, Lh and Fsh expression, and gametogenesis analysis for each oocyte stage. Repeated-measures ANOVA was used to determine significant differences in male and female reproductive outputs between genotypes. Student’s t-test was used to determine significant differences in Gnrh2 and Lh/Fsh interactions between fed and fasted pituitaries, expression differences between fed and fasted pituitaries or brains, and GSI levels between mutant and WT zebrafish in fed and fasted states. Statistical significance was determined as a P-value < 0.05.

## Data Availability

No datasets were generated or analyzed during the current study.
